# Real-world evaluation of a novel technology for quantitative simultaneous antibody detection against multiple SARS-CoV-2 antigens in a cohort of patients presenting with COVID-19 syndrome†
†Electronic supplementary information (ESI) available. See DOI: 10.1039/d0an01066a


**DOI:** 10.1039/d0an01066a

**Published:** 2020-07-07

**Authors:** Andrew M. Shaw, Christopher Hyde, Blair Merrick, Philip James-Pemberton, Bethany K. Squires, Rouslan V. Olkhov, Rahul Batra, Amita Patel, Karen Bisnauthsing, Gaia Nebbia, Eithne MacMahon, Sam Douthwaite, Michael Malim, Stuart Neil, Rocio Martinez Nunez, Katie Doores, Tan Kia Ik Mark, Adrian W. Signell, Gilberto Betancor, Harry D. Wilson, Rui Pedro Galão, Suzanne Pickering, Jonathan D. Edgeworth

**Affiliations:** a Department of Bioscience , College of Life and Environmental Sciences , University of Exeter , Stocker Road , Exeter , EX4 4QD , UK . Email: andrew.m.shaw@exeter.ac.uk; b Attomarker Ltd , Innovation Centre , University of Exeter , Rennes Drive , Exeter , EX4 4RN , UK; c Exeter Test Group , College of Medicine and Health , University of Exeter , St Luke's Campus , Heavitree Road , Exeter , EX1 2LU , UK; d Centre for Clinical Infection and Diagnostics Research , Department of Infectious Diseases , Guy's and St Thomas’ NHS Foundation Trust , London SE1 7EH , UK . Email: Jonathan.Edgeworth@gstt.nhs.uk; e Department of Infectious Diseases , School of Immunology & Microbial Sciences , King's College London , UK

## Abstract

An evaluation of a rapid portable gold-nanotechnology measuring SARS-CoV-2 IgM, IgA and IgG antibody concentrations against spike 1 (S1), spike 2 (S) and nucleocapsid (N) was conducted using serum samples from 74 patients tested for SARS-CoV-2 RNA on admission to hospital, and 47 historical control patients from March 2019. 59 patients were RNA(+) and 15 were RNA(–). A serum (±) classification was derived for all three antigens and a quantitative serological profile was obtained. Serum(+) was identified in 30% (95% CI 11–48) of initially RNA(–) patients, in 36% (95% CI 17–54) of RNA(+) patients before 10 days, 77% (95% CI 67–87) between 10 and 20 days and 95% (95% CI 86–100) after 21 days. The patient-level diagnostic accuracy relative to RNA(±) after 10 days displayed 88% sensitivity (95% CI 75–95) and 75% specificity (95% CI 22–99), although specificity compared with historical controls was 100% (95%CI 91–100). This study provides robust support for further evaluation and validation of this novel technology in a clinical setting and highlights challenges inherent in assessment of serological tests for an emerging disease such as COVID-19.

## Abstract 2

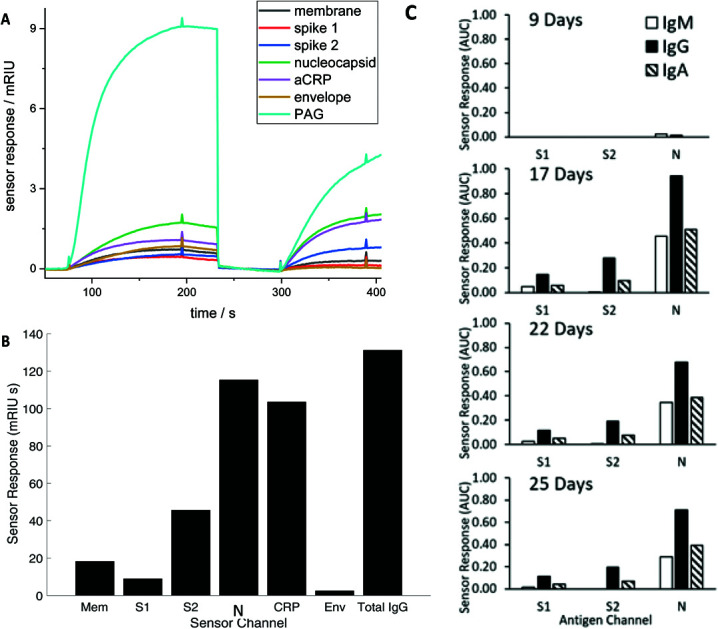
An evaluation of a rapid portable gold-nanotechnology measuring SARS-CoV-2 IgM, IgA and IgG antibody response to spike 1 (S1), spike 2 (S) and nucleocapsid (N) antigens using serum from 74 RNA(+) patients and RNA(+) 47 control patients.

## Introduction

Coronaviruses cause disease in birds and mammals^1^,^2^ and usually cause mild respiratory diseases in humans; however, strains have emerged such as SARS and MERS causing outbreaks of lethal respiratory disease^1^ and in December 2019 a novel coronavirus was identified in Wuhan, China. The causative agent named SARS-CoV-2 causes coronavirus disease 2019 (COVID-19) and has led to a global pandemic.

Patients presenting to hospital with clinical and radiological features consistent with COVID-disease usually have a SARS-CoV-2 RNA PCR test performed on upper respiratory tract specimens (*e.g.* nose and throat swabs) to confirm the diagnosis. Throughout this paper we refer to positive results as RNA(+) and negative as RNA(–). The reliability of PCR swabs are subject to pre-analytical errors such as the quality of sample collection, the technology platform and the primers designed, and for clinical reasons such as infection being localised to the lower respiratory tract.^3^ Some patients also present late when the viral infection may have passed when symptoms may predominantly be due to immunological, inflammatory and thrombotic processes.^4^ Comparisons between clinical, radiological and PCR findings illustrate these challenges. In one study 35% of patients with positive CT scan findings were admission RNA(–). Review of serial CT images and clinical findings showed 17% and 12% of admission RNA(–) patients were finally given a COVID-19 diagnosis, and 93% became RNA(+) after further testing over 5 days.^3^ These observations illustrate the benefit of aggregating information from multiple sources to support the clinical diagnosis from which the many management decisions can take place.

SARS-CoV-2 infection stimulates an antigen specific antibody response. Detecting these antibodies has potential to provide diagnostic information, even though serology is not conventionally used for diagnosis of acute respiratory viral infection such as influenza. Serology may also have a role in population screening, modelling disease spread in the community and staff surveillance, and there may be different required performance criteria in these different settings. There have been a number of reports describing SARS-CoV-2 antibody detection methodologies and technologies, including ELISA assays and lateral flow devices. None is currently considered to have acceptable sensitivity or specificity for diagnosis.^5^

Here we present a detailed evaluation of a novel gold nanoparticle array technology that provides a quantitative multiplexed 9-dimensional measure of the IgG, IgA and IgM response to SARS-CoV-2 S1, S2 and N proteins. The study was performed using a pre-determined set of samples obtained from a real-world cohort of patients admitted to St Thomas’ Hospital with a suspected clinical diagnosis of COVID-19 on admission and in whom a SARS-CoV-2 RNA PCR was performed. The results of the multiplexed response profile were related to RNA(±) patient classification and time. This robust initial analysis supports proceeding to validation of this technology as a potential serological technology solution for addressing key needs in response to the SARS-CoV-2 pandemic.

## Experimental methods

### Multiplexed COVID-19 antigen array and liscar reader

The tests were performed on the portable bench-top multiplexed array technology that has been described in detail elsewhere.^6^–^10^ It has been shown effective at detecting antibody in response to vaccination^11^ and has characterised accuracy and precision for CRP and total IgG assays^6^ with typically 10% accuracy and intra-day precision of less than 5%. The technology consists of an array of 170 of gold nanoparticle spots which scatter light into a video camera when illuminated from below (Fig. S1†). Each array includes antibody to capture CRP, Protein A/G to capture total Fc-binding antibodies and COVID-19 recombinant antigens S1, S2, and N protein along with SARS membrane (M) and envelope (E) proteins.

**Fig. 1:**
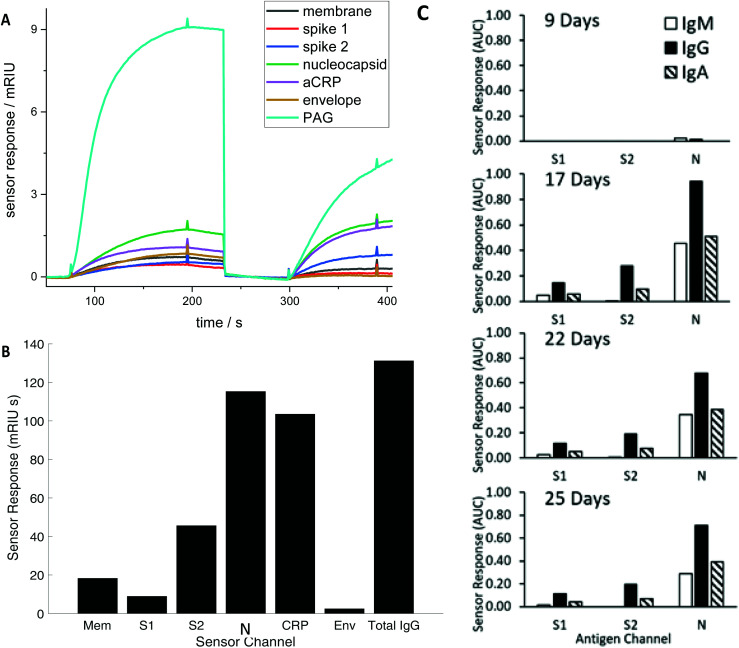
(A) Time course of the simultaneous evolving sensor response for each antigen in real time for a representative RNA(+) patient showing the combined IgM, IgG, and IgA response against membrane (Mem), spike (S) 1 and 2, nucleocapsid (N) and envelope (Env), and host proteins aCRP, total IgG (represented as the capture antigen Protein A/G (PAG)). The first set of responses (before 250 seconds) shows binding in response to patient serum exposure and the second set of responses the detection antibody (300–400 seconds) (B) The combined IgG, IgA and IgM sensor response in mRIU seconds is shown for each viral and host antigen integrated over 120 seconds. The total serum IgG level (represented as PAG in (A) is divided by 5 (C) Evolution of the IgM, IgG and IgA response for a single patient.

Diluted serum (or whole blood) is injected into the device to flow over the array producing a brightness change time (Fig. 1) during a loading step, before undergoing a wash cycle. A detection anti-CRP completes a sandwich assay for the CRP assay and an antibody recognising all human IgG, IgM and IgA detects total serum antibodies binding to each SARS CoV2 antigen (the Rapid Test). Alternatively, antibodies specific to IgG, IgM and IgA in series can be applied to detect individual responses to determine specific IgG, IgM and IgA against each antigen (The Antibody Class Differential Test). The light intensity change in the detection step is integrated for two minutes to produce an area under the curve (AUC) of light intensity seconds (Fig. 1). The test cycle takes 7.5 minutes followed by 2.5-minute regeneration.

### Array biofunctionalisation and materials

The arrays were functionalised according to the procedure detailed elsehwhere.^6^ Briefly, the antigens S1, S2, E/M antigens were printed as received from the supplier onto the gold surface functionalised with EDC/NHS. The N antigen was buffer exchanged prior to printing into the standard PBS/glycerol buffer. The arrays were washed and blocked with serum at stored at 4 °C prior to use.

The materials used in the course of the experiments were used without further purification, as certified by the suppliers. Sigma-Aldrich supplied phosphate buffered saline in tablet form (Sigma, P4417), phosphoric acid solution (85 ± 1 wt% in water, Sigma 345245), Tween 20 (Sigma, P1379), *N*-(3-dimethylaminopropyl)-*N*′-ethylcarbodiimide hydrochloride (commercial grade, E7750), *N*-hydroxysuccinimide (98%, 130 672) and bovine serum albumin (>98%, A3059). SAM molecules, linker HS-C_11_-EG_6_-OCH_2_-COOH (>95%, TH 003-M11.N6) and spacer HS-C_11_-EG_3_-OH (>95%, TH 001-M11.N3), were supplied by ProChimia Surfaces. Native human C-reactive protein (>99%, P100-0) and CRP depleted serum (SF100-2) were obtained from BBI Solutions. Glycine (analytical grade, G/0800/48) was provided by Fisher Scientific and recombinant PAG (21186) was supplied by ThermoFisher Scientific. The assay running and dilution buffer was PBS with 0.005 v/v % Tween 20 and the regeneration buffer was 0.1 M phosphoric acid and 0.1 M glycine solution in deionized water.

The recombinant antigens REC31754-100 SARS Coronavirus Envelope Protein (*E. coli*); REC31753-100 SARS Coronavirus Membrane Protein (Matrix) (*E. coli*); REC31806-100 SARS-CoV-2 Spike Glycoprotein (S1), SHFc-Tag (HEK293); REC31807-100 SARS-CoV-2 Spike Glycoprotein (S2), SHFc-Tag (HEK293); REC31812-100 SARS-CoV-2 Nucleoprotein His-Tag (*E. coli*); and PAB21446 Rabbit Anti-Sheep (H + L) were supplied by the Native Antigen Company (Oxford). SAB3700698 Anti-Sheep IgG (Fc specific) polyclonal Antibody produce in rabbit, sourced from Sigma-Aldrich.

Detection reagents were supplied by Bio-Rad, 5211-8004 Goat Anti-Human IgG polyclonal Antibody, STAR141 Goat Anti-Human IgA polyclonal Antibody, STAR145 Goat Anti-Human IgM polyclonal Antibody, STAR 125 Goat Anti-Human IgG polyclonal Antibody, 1707-0189G goat anti human CRP polyclonal antibody; and Randox: IA8354 Immunoturbidimetric Anti-Human IgA Antibody, IG8352 Immunoturbidimetric Anti-Human IgG Antibody, IM8353 Immunoturbidimetric Anti-Human IgM Antibody.

A serum negative standard, CRP depleted serum (BBI SF100-2) acting as CRP(–) control and a SARS-COV-2 antibody negative control. Similarly, a CRP positive control (Level 3 Randox CP2481) is a SARS-COV-2 antibody negative control. A positive patient control was identified early in testing and used as part of the QC process.

### Array test protocol

COVID-19 chips are calibrated using the CRP assay referenced to the WHO CRP standard material. A calibration curve for CRP has a lower limit of detection of 1 mg L^–1^ and a dynamic range to 150 mg L^–1^.^6^ The total antibody assay is calibrated using the NIST standard antibody to determine the absolute surface binding site density. Only the CRP and PAG assays can be calibrated absolutely as standard reference materials are available; no reference materials were available for the antigen antibodies at the time of data collection. The surface antigen channels are calibrated by an antibody raised to sheep Fc region synthesised on the recombinant S1 and S2 antigens or a poly-his-tag synthesised on the recombinant membrane antigen for purification. The tags are used or affinity purification but can also be used to determine the antigen binding site density on the surface which can be used to predict the calibration curve but allows the relative intensities to be estimated (semi) quantitatively. The error in the calibration process predicted from binding site density is associated with non-specific binding. The mean coefficient of variation across repeats with the same sample performed on the same arrays without any correction were S1, 7.8%, S2 19.2%, N 7.8%, CRP, 10% and Total IgG 1.5%. The Limit of Blank was 0.186 ± 0.07 (mean ± standard deviation) compared with the N(+) cut-off of 0.31.

A dilution study was performed on a positive patient to establish the antibody titre and CRP concentration (Fig. S2†). It shows that neither assay is saturated indicating a significant dynamic range and the samples with the 100-fold initial dilution are well placed on the calibration curve. Antibody titre is used as a measure of concentration when reference materials are not available; an *n*-fold dilution of a sample is performed until the signal falls below the detection limit. Relative concentration can then be determined from dilution and absolute concentrations by determining the titres from the NIST reference antibody of WHO CRP reference materials. for all antibody titres. The N antibody titre is 1/6700–1/10 000 compared with the CRP at 300 mg L^–1^ with titre 1/1000. Patient testing was performed under batch control with two refractive index standards to calibrate the brightness change, a serum negative standard, a CRP positive standard and a positive patient standard repeated every 20 patients (Fig. S2†). Degradation of the antigen chip was recorded for each batch under standardised conditions and varied between patient samples applied allowing between 30–50 sample runs per chip before replacement. Chip-to-chip variation was normalised using control samples.

### Study design

As a first assessment of the device clinical validity amongst hospitalized patients, the design was initially conceptualised as a two-gate diagnostic accuracy study^12^ of test performance in detecting COVID-19 infection, as defined by a positive SARS-CoV-2 RNA PCR swab during that admission. However, the design had to be modified to acknowledge that while a positive PCR test strongly indicates the presence of COVID-19 disease, a negative result is weakly indicative of the absence of disease because of well documented and theoretically plausible false negative PCR test results in a significant minority of patients. Further, index test negativity in PCR positive patients might be expected in patients in the early stage of disease before antibodies are produced or in patients who could not mounting an antibody for whatever reason. We thus focused on descriptively reporting findings of the index test in the patient sample series. We considered results at a sample level and a patient level, particularly evolution of results over time.

Viral RNA positivity or negativity at any stage during admission was retained as an important explanatory variable for results, alongside others, and percentage agreement calculated with 95% confidence intervals.^13^ Although these can be considered as early estimates of accuracy (sensitivity and specificity), great caution is required in their interpretation because of the underlying uncertainty about the true disease state, particularly absence of disease. Specificity is thus affected more than sensitivity. In order to avoid bias from setting cut-offs for index test positivity using the same samples as those used for evaluating accuracy, we initially separated samples into Phase 1 (training set) and Phase 2 (evaluation set). Phase 3 was further added using new samples not examined in Phases 1 and 2, to specifically improve the precision of our “sensitivity” estimate. The size of this was informed by a target sample size estimation of 45 patients assuming a true value of 100% and a lower 95% CI of 90%. The measurement of specificity relative to stored samples before the start of the COVID pandemic constituted a fourth phase of the investigation. The size of this was again informed by a sample size estimation of 45 patients assuming a true value of 100% and a lower 95% CI of 90%. Finally, to specifically extend the patient antibody profiles, additional specimens for these patients were purposively sought. These were not included in the analyses of any other phases. For the patient level analysis, where there are multiple samples for the patient, a summary value was calculated as the mean of the sample values, having first confirmed that there was consistency between the values, which was true in all patients. The results were reported according to the STARD reporting framework as the most relevant guideline for the study design undertaken.^14^

### Patient and sample origin

The cohort comprised patients admitted to St Thomas’ Hospital with suspected COVID-19 between February 27th and March 30th 2020 and on whom a SARS-CoV-2 RNA PCR test was performed. Surplus serum was retrieved from the routine biochemistry laboratory at point of discard, and then aliquoted, stored and linked with a limited clinical dataset by the direct care team, before anonymization under an existing ethics framework (REC reference 18/NW/0584) and with expedited R&D approval. Members of the research team conducting serology testing only had access to anonymised clinical information during the duration of the study. For Phases 1 and 2, although viral RNA status was known, no further clinical details were available to the analysing team prior to the device results being obtained. In Phase 3, viral RNA status and time of sample relative to symptom onset were known. Where the same patient contributed to multiple phases, it was with a different sample. 47 anonymised serum samples stored in March 2019 from patients who did not have had SARS-CoV2 infection stored for future technology evaluation projects under the same ethical approval as above, were used as a control cohort. The complete dataset will be fully anonymised at the end of the study period. All work was performed in accordance with the UK Policy Framework for Health and Social Care Research, and approved by the Risk and Assurance Committee at Guy's and St Thomas’ NHS Foundation Trust. Informed consents were not required from participants in this study as per the guidelines set out in the UK Policy Framework for Health and Social Care Research and by the registration with and express consent of the Institutional Review Board listed above.

## Results

### Patient and sample cohorts

Summary characteristics of all 119 samples used for the first 3 study phases collected from 74 patients admitted between 21/02/20 and 30/3/20 and tested for SARS-CoV-2 are summarised in Table 1. Distribution of samples and patients within each phase is shown in Table S1.† Where the same patient contributed to multiple phases, it was with a different sample. The cohort was typical of COVID-19 patients admitted to hospitals around the world with 27 patients admitted to the intensive care unit during their admission, and there had been 10 deaths and 25 patients discharged home as at 30/3/20. Recorded co-morbidities, outcomes and level of support for each patient linked with each serum sample is presented in Tables S1–3.† A statistically determined set of 47 stored serum samples from patients presenting to A&E before the start of the pandemic were used as a further control cohort.

**Table 1:** Summary patient cohort clinical characteristics

Cohort size	74
Age mean (SD)	55.3 (16.7)
Male	50 (68%)
No comorbidity	10 (14%)
Multiple comorbidities	48 (65%)
Mortality	10 (14%)
Discharged at time of analysis	21 (28%)
Median LOS not died or discharged (to 30/3/20) (range)	14 (0 to 38)
Required mechanical ventilation	38 (51%)
Required no respiratory support	14 (19%)
Viral RNA positive at any stage	59 (80%)
Single sample	61 (82%)
Multiple samples	13 (18%)
Mean number of samples where multiple	3.8

### Sample level analysis

Serum antibody testing against all antigens was evaluated on a single batch production of 6 COVID-19 chips each normalised for their refractive index sensitivity. A typical sensor response profile for each viral antigen and host protein (CRP and total IgG) is shown in Fig. 1(A) The combined IgG, IgA and IgM response against each viral antigen is shown in Fig. 1(B). The dominant combined IgM, IgA and IgG response is consistently observed against N with a range 100–300 mg L^–1^ for the samples tested. Individual quantitative IgM, IgA and IgG responses to each antigen were performed for a number of patients to determine the evolution of the differential IgM, IgG and IgA response over time, shown as a time course for one patient (Fig. 1C).

**Fig. 2:**
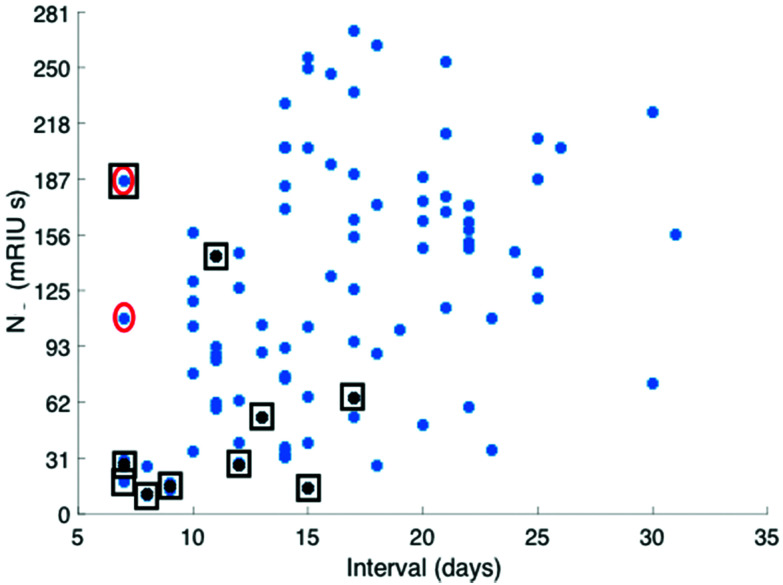
Distribution of total antibody response to N antigen (mRUI seconds) plotted against time from first day of patient reported symptom onset recorded by the admitting patients care team. Serum total antibody levels against the N antigen recorded from patients who had died at the end of the study period are identified with a black square (as of 30/3/20).

The combined IgM, IgA and IgG antibody response against N was taken forward as the serological response marker for SARS CoV-2 infection due to it having the highest quantitative response. The N antibody level detected in all 119 serum samples plotted against time from patient-reported first day of symptom onset is shown in Fig. 2. A sample classification was set at <10 days consistent with reported onset of serological response.^15^ Two serum samples taken from patients with <10 days of reported symptoms had high antibodies against N. Clinical data provided to the laboratory team were reviewed. The upper red circled data point is from a patient admitted with a differential diagnosis of community-acquired pneumonia. They had a negative SARS-CoV-2 nose and throat PCR performed on day 10 and day 18 of symptom onset and a negative PCR on a bronchoalveolar lavage taken on Day 21 while on ITU. The lower red circled data point was from a patient who had dementia and so the first day of symptom onset of interval may be incorrect.

**Fig. 3:**
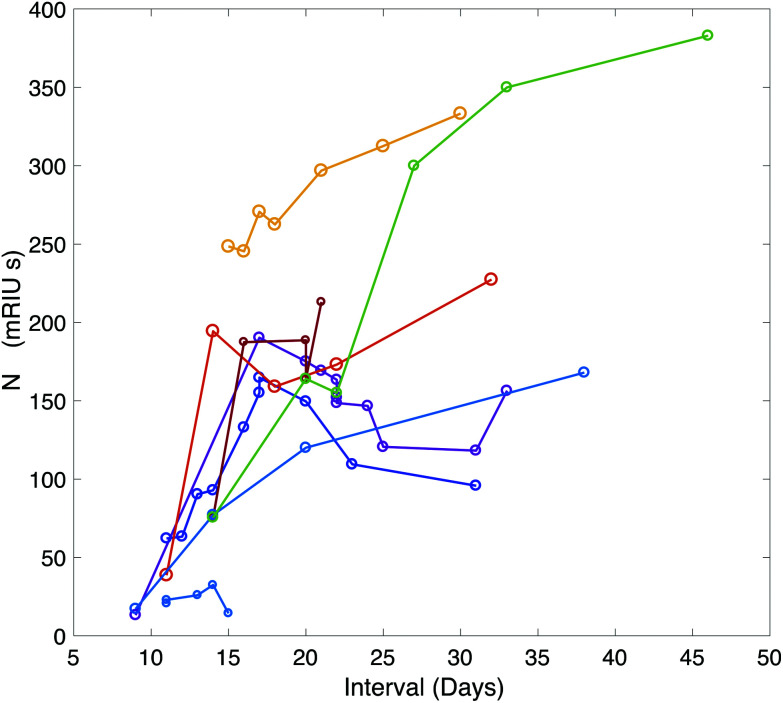
Time course of the antibody response against NC plotted over time for 9 patients during their hospital stay. Additional data points were added through analysis of serum samples provided by the clinical care team for patients who remained in hospital beyond the end of the study period.

A time-course of the antibody response against N was plotted for 9 patients using serum samples taken between symptom-day 9 and 45 (Fig. 3). This included serum samples provided from patients who remained in hospital beyond the end of the study period. One patient who died on Day 15 had negative N antibody measurements in 4 serum samples taken daily between day 11 and 15.

**Fig. 4:**
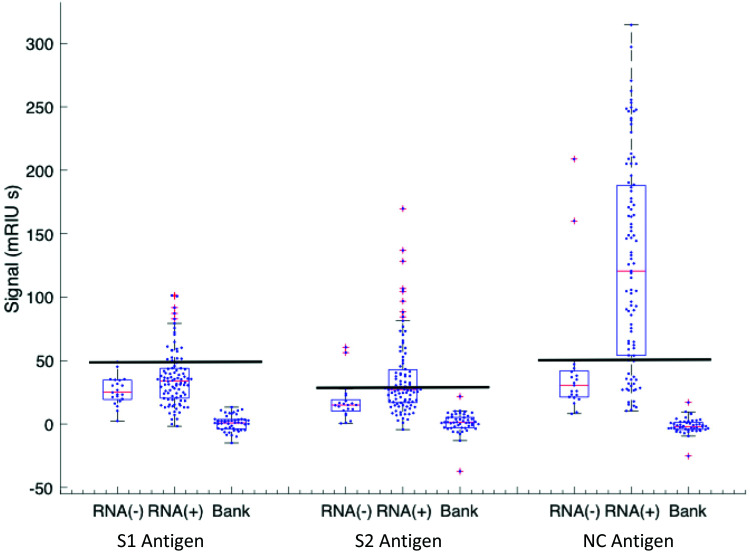
Antigen response for S1(3×), S2 (3×) and N response for RNA(–) and RNA(+) patients in the cohort. The RNA(–) shows outliers in all three antigen distributions and the cut-off is set at the highest value in the distribution (line). The RNA(+) patient group has varying intervals from onset of symptoms with varying rates of recovery. There is a set of patients who have not yet responded or not going to respond (below the cut-off line) that correspond to the early-time patients below 10 days shown in Fig. 1. These are excluded in the recovering +10 days cohort. The biobank data set is included for the specificity analysis.

Classification of a serum sample being either S1, S2 or N antibody positive was made based on RNA(–) phase 1 samples from which cut-off levels for each viral antigen were derived (see Methods). The antibody levels against S1, S2 and N for all samples taken from RNA(–) and RNA(+) patients with their respective cut-offs presented as horizontal black lines are shown in Fig. 4. A Boolean classifier of being S1(+) or S2(+) or N(+) was used to classify patients as seropositive, rather than using N antibody response data alone as presented in Fig. 2 and 3 (Table 2). Using this classification 7(30%) of RNA(–) samples had serum(+) antibody concentrations above the cut off for all three viral antigens (triple RNA(–) false negative). The triple classification assessment identified 22% of all serum positive samples as S1(+) S2(–) N(+), 51% S1(–) S2(+) and N(+) and 18% S1(+) S2(+) and N(+).

**Table 2:** Sample serum classification table using three-antigen Boolean classifier

	Serum (+)	Serum (–)	Total	Serum (+) percentage
RNA(–)	7	16	23	30% (95% CI 11–48)
RNA(+) all intervals	76	20	96	79% (95% CI 71–97)
Total	83	36	119	

Interval samples				
RNA(+) < 10 days	9	16	25	36% (95% CI 17–54)
RNA(+) 10–20 days	53	16	69	77% (95% CI 67–87)
RNA(+) > 21 days	21	2	23	95% (95% CI 86–100)
Total	83	34	117^*a*^	

^*a*^Time interval not available for two patients.

Classifications of all samples tested in phase 1, 2 and 3 are shown in Table 2 and show a 79% (95% CI 71–97) serum antibody positive rate for the whole clinically heterogenous cohort at all time intervals including the slow-response and patients who died early and those that did not appear to show an antibody response over time. The positivity rate varied by interval from day of first symptom onset with maximum sensitivity of 95% (95% CI 71–97) being observed at 21 days. Finally, a further 47 randomly selected anonymised serum samples from the diagnostic laboratory taken during March 2019 were provided by the clinical team for analysis, and all were serum(–) for all three antibodies against all three viral protein targets (Fig. 4).

### Patient level analysis

The patient-level analysis initially focused on the 24 patients evaluated in the second phase of testing. Using thresholds derived from the first phase of testing for S1(+) 0.10 (16 mRIUs), S2(+) 0.05 (8 mRIUs) and N(+) 0.31 (47 mRIUs), the sensitivity based on being (+) for any one antigen was 100% (95% CI 52–100) and specificity 75% (95% CI 22–99%) for samples taken ≥10 days after the onset of symptoms (see Table 3 upper). The third phase was designed to increase the precision of the sensitivity estimation. Additional serum samples from different time points were provided for 43 known RNA (+) patients, 49 in total. The revised sensitivity estimation was 88% (95% CI 75–95) (see Table 3 lower). Using stored samples from March 2019 the specificity was 100% (95% CI 91–100).

**Table 3:** Patients with samples ≥10 days post symptoms, contingency tables (threshold three-antigen Boolean classifier)

	Viral RNA(+)	Viral RNA(–)	
Phase 2			
S1 or S2 or N ≥ threshold	6	1	7
S1 or S2 or N < threshold	0	3	3
Totals	6	4	10

Phase 3			
S1 or S2 or N ≥ threshold	43		
S1 or S2 or N < threshold	6		
	49		

## Discussion and conclusions

This paper provides a robust, real-world assessment of the potential for a novel and rapid quantitative multiplexed gold nanoparticle technology detecting antibodies against SARS-CoV-2 antigens to assist with clinical diagnosis and decision making. It was performed on a cohort of 74 patients admitted to hospital early in the pandemic and suspected of having serious COVID-19 disease. The patient cohort was clinically heterogeneous and characteristic of any scenario in which a new antibody test must be validated, rather than being a carefully selected test cohort that may introduce sources of bias that provide artificially favourable technology evaluations.

Responses were seen to N, S1 and S2 proteins that were above derived threshold settings. The highest antibody titre was against N and so N responses were used to inform setting the time cut-off classification and to create serological response profiles for 9 patients. The response profiles showed the technology was capable of reproducibly detecting quantitative antibody levels over time on different samples. In contrast only 18% of samples showed a positive result for all three antigens (S1, S2 and N) with 50% having N and S2 responses but only 22% N and S1 responses. The RNA(–) false negative rate was estimated to be 30% (95% CI 10–46) which is consistent with findings elsewhere^16^ although sensitivity of PCR assays may improve with refinement of primers and technologies.^17^ Serum positivity was defined as having an antibody response above cut-off against any one of the three antigens, such that 30% (95% CI 11–48) of initially RNA(–) patients were identified as serum positive. For samples taken from RNA(+) patients, the positivity rates were 36% (95% CI 17–54) before 10 days, 77% (95% CI 67–87) between 10 and 20 days and 95% (95% CI 86–100) after 21 days. The patient-level diagnostic accuracy relative to RNA(±) after 10 days gave a sensitivity of 88% (95% CI 75–95) and specificity 75% (95% CI 22–99), although specificity compared with historical controls was 100% (95%CI 91–100).

Our findings are consistent with other published studies^15^,^16^,^18^–^25^ up to 9/4/20. The main evaluation approach in most studies has been to identify patients with confirmed RNA(+) COVID-19 disease (“cases”) and then compare results with “controls”, which are either healthy normal patients or less commonly patients with suspected COVID-19, but in which disease had been excluded.^21^,^22^ Results are then measured in each group to give estimates of sensitivity from results classification in the “cases” and specificity from results classification in the “controls”. Overall sensitivity of 89% and specificity of 91% found by Li *et al.*^21^ was typical of the favourable evaluations. The estimate in the unfavourable evaluation was sensitivity 14% and specificity 92%.^16^ However, time interval since onset of symptoms is an obvious variable^15^ and most studies thus far have not taken this into account in their accuracy estimates, including that from the unfavourable evaluation, acknowledging that this can be hard to precisely define from patient memory and perception of disease symptomatology.

Other challenges which were often not met include recognising the implications of different reference standards, separating sample and patient levels of analysis and giving measures of uncertainty. We identified only one study^16^ which attempted to look at classification of real-world populations, where patients had not been specifically pre-separated into “cases” and “controls”. Cohort heterogeneity is a clear and important challenge in any clinical setting. We tried to overcome these challenges in our study, particularly by setting out on our assessment of this technology by first measuring antibody responses in a clinically relevant diverse patient cohort. However, we had to rely on sensitivity and specificity measured in cases and controls in subsequent phases to make the most of the available samples, which is a limitation. Difficulty blinding investigators to the nature of the sample is a further limitation for this component of our study, but was not true where the initial mixed clinical cohort was investigated. Defining target condition by a composite reference standard including clinical profile as well as viral-RNA would have improved our study too. We would have liked to look in more detail at the relationship between false positives and false negatives and clinical characteristics, but these were small in number and our ethics did not permit such detailed clinical evaluations. Concerning the size of the study generally, although the number of patients and samples were modest, the numbers we analysed were guided by sample size calculations. We acknowledge that future studies need greater numbers to further improve precision.

For future research, two-gate designs (the preferred name for diagnostic case-control accuracy studies) should be avoided. Two-gate designs calculate sensitivity in those who definitely have the condition and specificity in those who definitely do not have the condition, at its extreme completely healthy people. Although they are a pragmatic approach, often employed early in the development of a test, they have been shown to exaggerate the performance of tests^26^,^27^ by excluding borderline cases which will actually make up a high proportion of patients when the test is actually used in practice. The problem arising from use of a two-gate design may be regarded as an extreme form of spectrum bias, a major concern in the test evaluation.^28^–^30^ For these reasons, evaluation is preferred in a realistic spectrum of disease in population where one expects the test to be used with a naturally occurring prevalence and spectrum of disease.

Since we conducted the literature review to inform our study, other influential evaluations of antibody tests have emerged. That by the National COVID Testing Scientific Advisory Panel in the UK has attracted particular attention. They concluded that the performance of current lateral flow immunoassay devices was inadequate for most individual patient applications. However, we observe that our device is different to the lateral flow immunoassay devices^5^ and performed similarly to the novel ELISA tested: sensitivity against an RT-PCR-confirmed diagnosis of SARS-CoV-2 infection 85% (95%CI 70–94); specificity *vs.* pre-pandemic controls 100% (95%CI 93–100). They used similar methods and sample sizes to this study. We are currently testing samples from a cohort of known RNA positive and RNA negative patients to assess against the sensitivity and specificity requirements set by regulators.

## Funding

The testing and our follow up research has been funded by over 1000 benefactors who have donated to the University of Exeter emergency appeal to support the work of Prof Shaw. The research was funded/supported by the National Institute for Health Research (NIHR) Biomedical Research Centre based at Guy's and St Thomas’ NHS Foundation Trust and King's College London, programme of Infection and Immunity (RJ112/N027). AWS was supported by the MRC-KCL Doctoral Training Partnership in Biomedical Sciences (MR/N013700/1); GB was supported by the Wellcome Trust (106223/Z/14/Z to MHM); PM, SP and HW were supported by the Wellcome Trust (WT098049AIA to SJDN and Chad Swanson).

## Conflicts of interest

Professor Shaw is CEO and Founder of Attomarker Ltd and has a shareholding. Dr Rouslan Olkhov has a shareholding in Attomarker. Philip James-Pemberton is a PhD student sponsored by Attomarker. Attomarker owns the Intellectual Property for the array reader technology. Professors Edgeworth and Hyde, and all other authors have no conflict of interest in Attomarker Ltd or any further interests to declare.

## Supplementary Material

Supplementary information
