# Supplementary material for: Real-world evaluation of a novel technology for quantitative simultaneous antibody detection against multiple SARS-CoV-2 antigens in a cohort of patients presenting with COVID-19 syndrome
Source: Analyst. 2020 Jul 7;145(16):5638–46. doi: 10.1039/d0an01066a (PMC7953841; doi:10.1039/d0an01066a)
Supplement: Supplementary file 1 [file AN-145-D0AN01066A-s001.pdf]

Electronic Supplementary Material (ESI) for Analyst.
This journal is © The Royal Society of Chemistry 2020

Supplementary tables

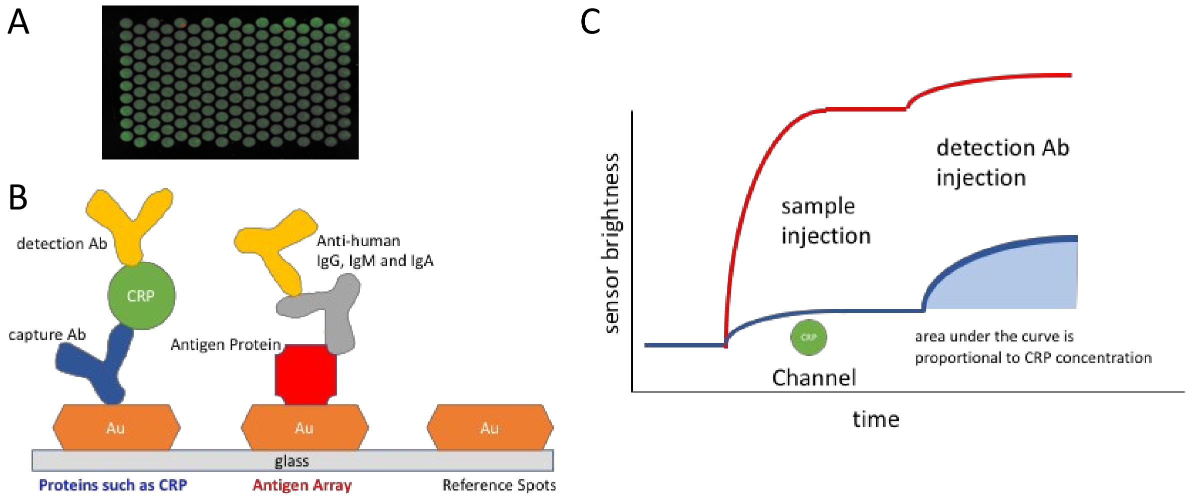

Figure S1 Assay principles: (A) Picture of a single chip with an array of 170 gold spots each adapted for different capture assays (actual size 3mm by 4mm (B) Representation of the target antigen capture assays. The CRP assay has a capture antibody bound to the gold particle spot and the SARS-CoV2 assays have viral proteins bound to gold particles. Serum at 1 in 100 dilution) is passed over the chip to capture IgG, IgM or IgA against each viral protein or the target host protein and then a detection antibody is added to complete a sandwich assay; (C) changes in brightness of the spots are detected at each incubation step which is measured as area under the curve (AUC) and converted into a quantified response

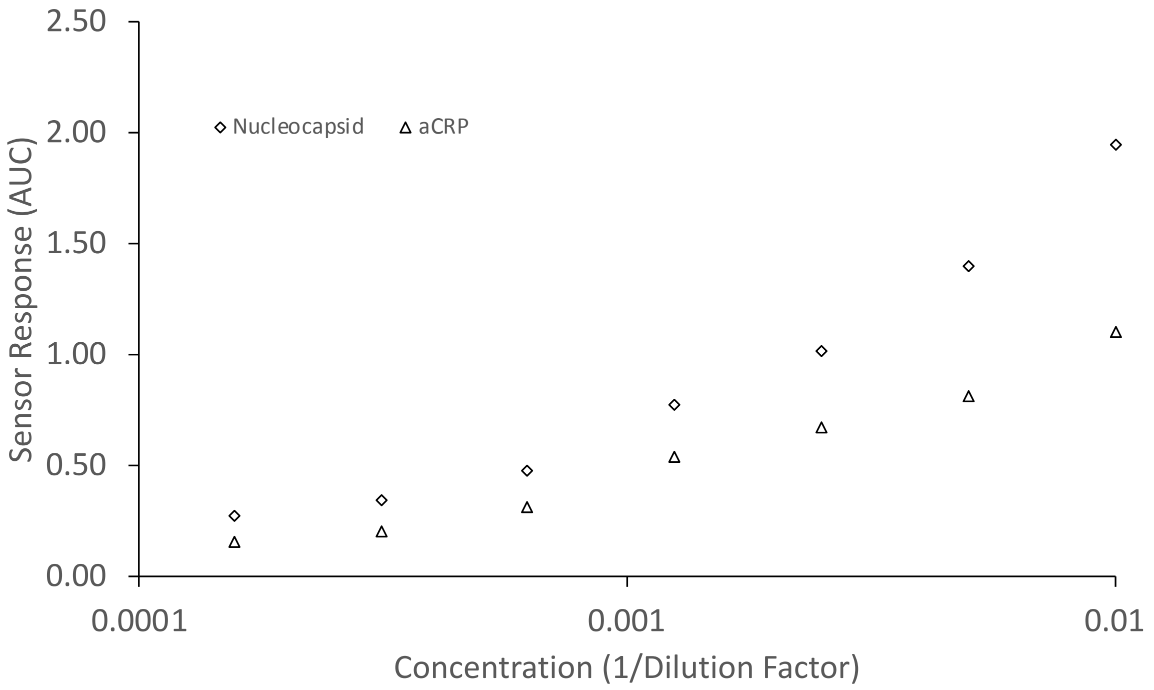

Figure S2 N antibody and CRP dilution studies for a N(+) patient. Data used to establish the antibody titre of 1/6700

Table S1 Sample and patient disposition for each phase

	Phase 1	Phase 2	Combined 1 & 2	Phase 3 *	All**
Analyses	16	67	83	84	167
Analyses on patient samples	15	49	64	55	119
Analyses on volunteer samples	0	1	1	0	1
Control and calibration analyses	1	17	18	29	47
Patients	10	24	31***	53****	74
11 samples/patient					1
10 samples/patient		1	1		
9 samples/patient					1
8 samples/patient			1		1
7 samples/patient		1	1		
6 samples/patient		1			
4 samples/patient			1		3
3 samples/patient	1		1		1
2 samples/patient	3	5	6	2	9
1 samples/patient	6	16	20	51	58

Note: * Serum sample from RNA(+) with an interval more than 10 days after onset of symptoms<br>** There were 47 further analyses performed on stored samples prior to the onset of the COVID pandemic to calculate best case specificity.<br>*** 3 different samples were analysed in both Phase 1 and Phase 2 from the same patients<br>**** 43 new patients. 1 patient was analysed in Phases 1 & 3, 7 patients were analysed in Phases 2 & 3, and 2 patients were analysed in all phases, with no samples being repeated between phases					

Table S2: Clinical characteristics of the 74 patients used for all three phases Clinical data was obtained as written by the admitting clinical team from the electronic notes and therefore data may be incomplete, inaccurate and were not refined further prior to linkage with the serum sample and anonymisation

Patient ID	Age	Sex	Comorbidities	Admitted to ITU	Max level of respiratory support	Outcome (as of 30/3/20)
1	46	M	Asthma, anxiety	Y	ECMO	ITU
2	34	F	Depression	Y	ECMO	ITU
3	66	M	None	Y	ECMO	HDU
4	28	F	ESRF, renal transplant	Y	MV	ITU
5	67	M	HTN, hypercholesterolemia, T2DM	Y	MV	Ward
6	48	F	None	N	None	Discharged
7	64	F	Depression, HTN, OA, OSA	Y	MV	ITU
8	56	M	COPD, ETOH xs, OSA, TIA	N	Supplemental O_2	Discharged
9	51	F	Asthma, obesity	Y	MV	ITU
10	48	M	Fibromyalgia	N	None	Discharged
11	92	M	AF, bladder ca, COPD, dementia, MGUS	N	Supplemental O_2	Died
12	66	M	None	N	None	Discharged
13	64	M	bronchiectasis, hiatus hernia, T2DM	Y	MV	Died
14	47	M	ESRF, HTN, renal transplant, T2DM	N	None	Discharged
15	38	M	HIV	N	None	Discharged
16	74	M	GORD, HTN	Y	MV	Died
17	50	M	Ankylosing spondylitis	N	None	Discharged
18	77	M	HTN, hypercholesterolemia, PMR, prostate cancer	Y	MV	Died
19	44	F	CTD, ILD, T2DM	N	Supplemental O_2	Discharged
20	61	F	ESRF, HTN, T2DM	Y	MV	Ward
22	48	M	Autism, learning disability,	Y	MV	Ward
23	90	M	Heart failure	N	None	Died
24	33	F	ESRF, HTN, renal transplant, SLE,	N	Supplemental O_2	Ward
25	53	M	HTN, obesity, T2DM,	Y	MV	Ward
26	55	F	Asthma, CVA, dementia, IHD, obesity, RA	Y	NIV	Discharged
27	59	M	HTN, obesity, T2DM	Y	MV	Discharged
28	77	M	HTN, prostate cancer	Y	MV	ITU
29	70	M	Hypothyroidism	Y	MV	ITU
30	29	M	None	N	None	Discharged
31	71	M	HTN, hyperthyroidism, T2DM	Y	MV	Died
32	49	M	ALD, depression, IVDU	Y	MV	Ward
33	60	M	HTN, renal transplant, T2DM	N	None	Discharged
34	45	F	ILD	N	None	Discharged
35	72	F	HTN, hypercholesterolaemia, obesity, T2DM	Y	NIV	Died
36	49	M	Asthma, prostate cancer	Y	MV	ITU
37	81	M	Hypercholesterolaemia	Y	MV	Discharged
38	73	F	HTN, IHD, cancer (unknown primary)	N	Supplemental O_2	Discharged
39	52	F	CKD, Phaeochromacytoma, sickle cell disease	N	None	Discharged
40	34	F	Asthma	Y	ECMO	ITU
41	33	M	None	Y	MV	ITU
42	55	M	HTN	Y	ECMO	ITU
43	34	M	Asthma, obesity	Y	MV	ITU
44	44	F	Obesity	Y	MV	ITU
45	43	F	ESRF, HF, T1DM	N	Supplemental O_2	Died
46	63	M	T2DM, VTE	N	Supplemental O_2	Discharged
47	80	M	ARMD, HTN, T2DM	Y	MV	Died
48	22	M	Obesity, T2DM	Y	MV	ITU

49	72	M	T2DM	Y	MV	ITU
50	61	M	Hepatitis C, Huntingdon's disease, OP	N	Supplemental O_2	Ward
51	78	M	Dementia, HTN, rectal cancer, stroke, mycosis fungoides, hypothyroidism, hypercholesterolaemia	N	Supplemental O_2	Discharged
52	22	F	T2DM, Grave's disease	N	Supplemental O_2	Discharged
53	52	M	Marfan's syndrome, OSA, AF, MVP	N	Supplemental O_2	Discharged
54	23	F	ESRF, T1DM	N	Supplemental O_2	Discharged
55	47	M	None	Y	MV	ITU
56	49	M	HTN, CKD, obesity, hypothyroidism, GORD, gout	Y	MV	ITU
57	49	M	None	Y	MV	ITU
58	50	M	None	Y	MV	ITU
59	62	M	Asthma	Y	MV	ITU
60	60	M	T2DM, pancreatitis	Y	MV	ITU
61	57	M	None	Y	MV	ITU
62	68	M	HTN, T2DM	Y	MV	ITU
63	74	M	HTN, IHD	Y	MV	ITU
64	60	F	OA	Y	MV	Died
65	49	M	HTN	Y	MV	ITU
66	62	F	ESRF, HTN, hyperparathyroidism, SLE	Y	MV	ITU
67	28	M	None	N	None	Discharged
68	65	M	HTN, T2DM	N	Supplemental O_2	Ward
69	64	F	HTN, hypercholesterolaemia, stroke, T2DM	N	None	Discharged
70	81	M	Dermatitis, HTN, hypercholesterolaemia	N	Supplemental O_2	Ward
71	88	F	HTN, PMR, T2DM	N	Supplemental O_2	Ward
72	40	M	Obesity, T2DM	Y	MV	Discharged
73	61	F	ESRF, T2DM, HTN	Y	MV	Ward
74	38	F	Asthma, Hep C, HIV, VTE, BPD, EtOH excess	N	None	Discharged
75	37	M	EtOH excess	Y	MV	ITU

Abbreviations:

AF	Atrial fibrillation
ALD	Alcoholic liver disease
ARMD	Age-related macular degeneration
CKD	Chronic kidney disease
COPD	Chronic obstructive pulmonary disease
CVA	Cerebrovascular accident (stroke)
ECMO	Extracorporeal membrane oxygenation
ESRF	End-stage renal failure
ETOH excess	Alcohol excess
GORD	Gastro-oesophageal reflux disease
HTN	Hypertension
IHD	Ischaemic heart disease
ILD	Interstitial lung disease
IVDU	Intra-venous drug use
MGUS	Monoclonal gammopathy of undetermined significance
MVP	Mitral valve prolapse
MV	Mechanical ventilation
NIV	Non-invasive ventilation
OA	Osteoarthritis
OP	
OSA	Obstructive sleep apnoea
PMR	Polymyalgia rheumatica
SLE	Systemic lupus erythematosus
RA	Rheumatoid arthritis
T1DM	Type 1 diabetes mellitus
T2DM	Type 2 diabetes mellitus
TIA	Transient ischaemic attack
VTE	Venous thromboembolism

Table S3. Phase 2 patient-level N serum antibody levels ordered by size

Patient no	Sample base	Summary N antibody level (AUC)	RNA viral status	Summary sample day post symptoms	Day post admission	Location (30/3/20)	Max support
30	1	0.059168	-	13		Home	None
22	1	0.066018	+	8	15	Ward	MV
23	1	0.070705	+	8		Died	None
28	1	0.110129	+	9	17	ITU	MV
16	1	0.114642	+	7		Died	MV
8	2	0.123583	-	12		Home	SO_2
24	1	0.125009	-	6	38	Ward	SO_2
4	4	0.163166	-	13	23	ITU	MV
19	1	0.169999	+	8		Home	SO_2
29	1	0.170744	+	7	13	ITU	MV
31	1	0.176996	-	8	17	Died	MV
27	1	0.192014	+	7		Home	MV
10	2	0.203309	-	1	12	Ward	None
26	1	0.224497	-	4		Home	NIV
32	1	0.236412	+	7	27	Ward	MV
17	1	0.244243	-	No symptoms		Home	None
2	8	0.728324	+	15	22	ITU	ECMO
9	2	0.937441	+	21	20	ITU	MV
1	9	1.022914	+	22	32	ITU	ECMO
20	2	1.179993	-	18	27	Ward	MV
18	1	1.193415	+	7		Died	MV
5	3	1.206743	+	20	23	Ward	MV
3	7	1.580750	+	18	27	HDU	ECMO
25	1	1.635506	+	15	21	Ward	MV

Abbreviations:
N – Nucleocapsid; RNA – ribonucleic acid; ITU – intensive therapy unit; HDU – high dependency unit; MV – mechanical ventilation; SO_2 – supplemental oxygen; NIV – non-invasive ventilation; ECMO – extracorporeal membrane oxygenation

Table S4. Phase 3 N serum antibody levels in samples taken from patients ≥ 10 days post symptom onset and identified as being viral RNA positive. Samples are ordered by total N antibody level

Patient no	Sample base	N antibody level (AUC)	RNA viral status	Sample day post symptoms	Day post admission
38	1	0.172651	+	18	
35	1	0.173858	+	12	
55	1	0.182167	+	12	4

39	1	0.203238	+	14	
69	1	0.204497	+	14	
67	1	0.223491	+	10	
33	1	0.224285	+	14	
34	1	0.228217	+	23	
70	1	0.238411	+	14	2
71	1	0.254323	+	12	17
50	1	0.318333	+	20	26
45	1	0.345087	+	13	
65	1	0.346535	+	17	6
51	1	0.376382	+	11	
49	1	0.382171	+	22	7
47	1	0.41449	+	17	
52	1	0.418779	+	15	
43	1	0.466594	+	30	14
36	1	0.483158	+	14	17
28	1	0.493428	+	14	17
63	1	0.502228	+	10	4
68	1	0.570193	+	11	3
48	1	0.573723	+	18	14
46	1	0.616692	+	17	
72	1	0.658982	+	19	
53	1	0.669736	+	15	
59	1	0.671561	+	10	4
37	1	0.676574	+	13	
60	1	0.761472	+	10	4
62	1	0.804641	+	17	5
54	1	0.809546	+	12	
61	1	0.832908	+	10	4
42	1	0.865413	+	25	17
64	1	0.923203	+	11	
58	1	0.934913	+	12	3
57	1	1.00777	+	10	3
66	1	1.093049	+	14	12
7	2	1.153463	+	20	20
41	1	1.25281	+	16	8
44	1	1.311853	+	15	14
56	1	1.312048	+	26	14
29	1	1.312545	+	14	13
40	1	1.471366	+	14	16
